# Gaseous air pollution and emergency hospital visits for hypertension in Beijing, China: a time-stratified case-crossover study

**DOI:** 10.1186/1476-069X-9-57

**Published:** 2010-10-05

**Authors:** Yuming Guo, Shilu Tong, Shanshan Li, Adrian G Barnett, Weiwei Yu, Yanshen Zhang, Xiaochuan Pan

**Affiliations:** 1 School of Public Health and Institute of Health and Biomedical Innovation, Queensland University of Technology, Kelvin Grove, Brisbane, Queensland 4059, Australia; 2 Department of Child and Maternal Health and Institute of Child and Adolescent Health, Peking University School of Public Health, Beijing 100191, PR China; 3 Department of Environmental Pollution and Health, Chinese Research Academy of Environmental Sciences, Beijing 100012, PR China; 4 Department of Occupational and Environmental Health, Peking University School of Public Health, Beijing 100191, PR China

## Abstract

**Background:**

A number of epidemiological studies have been conducted to research the adverse effects of air pollution on mortality and morbidity. Hypertension is the most important risk factor for cardiovascular mortality. However, few previous studies have examined the relationship between gaseous air pollution and morbidity for hypertension.

**Methods:**

Daily data on emergency hospital visits (EHVs) for hypertension were collected from the Peking University Third Hospital. Daily data on gaseous air pollutants (sulfur dioxide (SO_2_) and nitrogen dioxide (NO_2_)) and particulate matter less than 10 μm in aerodynamic diameter (PM_10_) were collected from the Beijing Municipal Environmental Monitoring Center. A time-stratified case-crossover design was conducted to evaluate the relationship between urban gaseous air pollution and EHVs for hypertension. Temperature and relative humidity were controlled for.

**Results:**

In the single air pollutant models, a 10 μg/m^3 ^increase in SO_2 _and NO_2 _were significantly associated with EHVs for hypertension. The odds ratios (ORs) were 1.037 (95% confidence interval (CI): 1.004-1.071) for SO_2 _at lag 0 day, and 1.101 (95% CI: 1.038-1.168) for NO_2 _at lag 3 day. After controlling for PM_10_, the ORs associated with SO_2 _and NO_2 _were 1.025 (95% CI: 0.987-1.065) and 1.114 (95% CI: 1.037-1.195), respectively.

**Conclusion:**

Elevated urban gaseous air pollution was associated with increased EHVs for hypertension in Beijing, China.

## Background

Numerous recent studies have assessed the adverse effects of air pollution on population health, including mortality, hospital admissions, and emergency hospital visits (EHVs) for cardiovascular diseases, respiratory diseases, and other diseases [1-4]. Some studies found an elevated level of ambient air pollution increased the risks of mortality and morbidity [5,6], while others found inconsistent results [7]. Also, many studies focused on the relationship between ambient air pollution and subgroups of cardiovascular diseases such as coronary disease [2,8], arrhythmia [5,9], myocardial infarction [10], and heart failure [11,12]. Our previous studies found a positive association between particulate air pollution and EHVs for hypertension and cardiovascular diseases [1,13]. However, less evidence is available to illustrate the effect of gaseous air pollution on acute events for hypertension.

Hypertension is not only one of the most serious risk factors for deaths and disease worldwide [14], but is also a major contributor to chronic heart failure, and a major risk factor for stroke and coronary heart disease, and their progression [15]. In the United States, the number of deaths caused by hypertension rose by 53% from 1991 to 2001 [16]. Ninety-one percent of people with heart failure had preceding hypertension, and half of all patients suffering a heart attack (and two-thirds of those having a first time stroke) have a blood pressure greater than systolic blood pressure (SBP) 140 mmHg and diastolic blood pressure (DBP) 90 mmHg [16]. Research conducted in Beijing, China shows that about 47% of investigated people had hypertension [17]. It is important to identify triggers and/or risk factors for hypertension. Air pollution may induce hypertension, so it is necessary to examine the relationship between air pollution and hypertension.

A few studies have examined the relationship between gaseous air pollution and blood pressure. Ibald-Mulli et al. [18] carried out a study using a random population sample to assess the association between air pollution and blood pressure, and found that an increase of 80 μg/m^3 ^in SO_2 _was linked with an increase in SBP of 0.74 mmHg (95% CI: 0.08-1.40). De Paula Santos et al. [19] found SO_2 _had positive and statistically significant effects on blood pressure. SO_2 _was associated with blood pressure in cold weather, as well as NO_2 _in warm weather [20]. In addition, SO_2 _and NO_2 _also strengthened the association between particulate matter less than 2.5 μm in aerodynamic diameter and blood pressure [21].

In this study we used a time-stratified case-crossover design to examine the relationship between gaseous air pollution and EHVs for hypertension.

## Materials and methods

### Data on emergency hospital visits

Data on EHVs for hypertension were collected between Jan 1, 2007 and Dec 31, 2007 from the Peking University Third Hospital, located in the northwest of urban city of Beijing [1]. EHVs were coded according to the International Classification of Disease, tenth revision (ICD-10) for hypertension (ICD-10: I10). The primary diagnoses were used in this study. The EHVs for hypertension were diagnosed by the visits' symptoms, inquiries, and medical inspections so that we expect the misclassification rate to be relatively small. About 95% of visits for hypertension are diagnosed only with hypertension, while 5% have accompanying heart failure, myocardial infarction or other diseases. The cases in this study lived in the residential areas around the hospital or in the urban area in Beijing.

### Data on air pollution and weather condition

We accessed daily data on urban sulfur dioxide (SO_2_), nitrogen dioxide (NO_2_), and particulate matter less than 10 μm in aerodynamic diameter (PM_10_) from the Beijing Municipal Environmental Monitoring Center. Air pollutants were monitored at eight fixed monitoring sites which were distributed in the urban area of Beijing. The site map was shown in our previous study [1]. Hourly pollutant data was recorded at each site, which we made into daily averages at each site and then for the whole city. If there were missing data from a monitoring station on a given day, then the values from the remaining monitors were used to calculate the average concentration.

Daily data on temperature and relative humidity for the study period were obtained from the China Meteorological Data Sharing Service System.

### Data analysis

Spearman's correlation coefficients were used to evaluate the inter-relations between air pollutants and weather conditions. The time-stratified case-crossover design was used to analyse the association of gaseous air pollution and EHVs for hypertension. The case-crossover design compares the exposure in the case period when events occurred with exposures in nearby control periods to examine the differences in exposure which might explain the differences in the daily number of cases. In this study, the cases and controls were matched by day of the week to control for any weekly patterns in deaths or pollution. Controls were compared with cases using the time-stratified method with twenty-eight days strata. So the first stratum was Jan 01 to Jan 28, 2007, the second stratum was Jan 29 to Feb 25. For an EHV on Jan 31 the control days were Feb 7, 14 and 21. The case-crossover method controls for any long-term trends and seasonal patterns in hospital visits and air pollutants. Each case day had three matching control days. The model gives the odds ratio (OR) of an EHV due to an increase in air pollution. When using the case-crossover design, confounders related to individual characteristics such as age, sex, and education are inherently controlled for. Studies have demonstrated that the case-crossover gives unbiased estimates in the presence of strong seasonal confounding [22,23].

There may be a delay between exposure to pollution and onset of hypertension. To examine the hazard period of air pollution for hypertension, we used the polynomial distributed lag model to evaluate the possibly delayed effect of air pollutants [24]. The polynomial smoothing with four degrees of freedom for SO_2 _and NO_2 _was used in the models. The hazard period was defined the same day as the hospital visit to up to five days prior.

Daily data on temperature and relative humidity were included in all models as confounders [25], using the same polynomial spline as SO_2 _and NO_2_. Single pollutant and multiple pollutants models were used to control the influence of other air pollutants. ORs and confidence intervals (CIs) were calculated for each air pollutant. All statistical tests were two-sided. The "season" package of R (version 2.10.1) was used to fit the time-stratified case-crossover [26,27].

## Results

There were 1,491 EHVs for hypertension in Peking University Third Hospital during the study period. The descriptive statistics for air pollutants and weather conditions are shown in Table 1. The average concentrations of SO_2_, NO_2 _and PM_10 _were 47.5 μg/m^3^, 66.6 μg/m^3 ^and 149.3 μg/m^3^, respectively. The average levels of NO_2 _and PM_10 _air pollution were higher than the national secondary ambient air quality standard in China (40 μg/m^3 ^and 100 μg/m^3^). The average concentration of SO_2 _was lower than the national secondary ambient air quality standard in China (60 μg/m^3^). The average temperature and relative humidity were 14.1°C and 54.2%, respectively.

**Table 1:** Summary statistics of daily air pollutants, weather condition and emergency hospital visits for hypertension in Beijing, 2007

	Minimum	25%	50%	75%	Maximum	Mean	SD
SO_2 _(μg/m^3^)	6.0	15.0	26.0	64.0	247.5	47.3	48.6

NO_2 _(μg/m^3^)	17.6	51.2	64.0	78.4	150.4	66.6	22.8

PM_10 _(μg/m^3^)	15.0	96.0	140.0	184.0	600.0	149.3	85.7

Temperature (°C)	-6.1	3.7	14.5	25.0	30.7	14.1	10.7

Humidity (%)	15.0	37.0	54.0	74.0	97.0	54.2	20.9

EHVs	0	2	4	5	19	4.1	0.1

**Figure 1:**
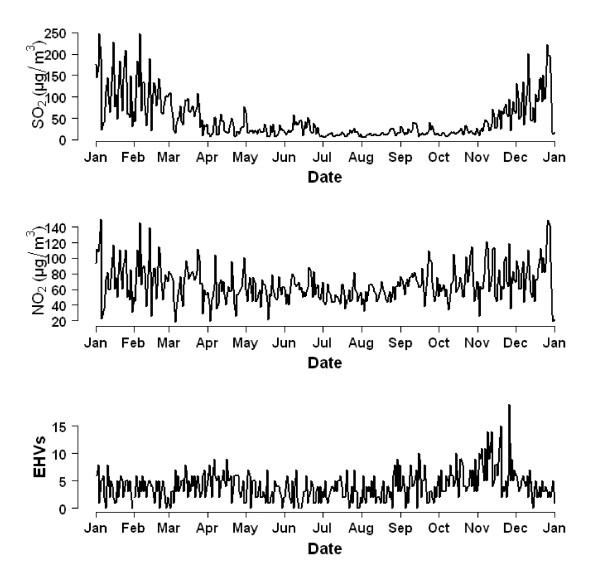
**The time series of daily mean concentrations of gaseous air pollutants and number of emergency hospital visits for hypertension in Beijing during 2007**.

Figure 1 shows the time series of gaseous air pollutants and EHVs for hypertension. The concentrations of both SO_2 _and NO_2 _were highest in winter. The number of EHVs for hypertension was highest in November.

Table 2 shows the correlations between air pollutants, temperature and humidity. PM_10_, SO_2 _and NO_2 _were significantly correlated with each other, e.g., between SO_2 _and NO_2 _(r = 0.65, P < 0.01), PM_10 _and SO_2 _(r = 0.46, P < 0.01), and PM_10 _and NO_2 _(r = 0.64, P < 0.01).

**Table 2:** Spearman's correlations between daily air pollutants and weather conditions in Beijing 2007

	PM_10_	SO_2_	NO_2_	Temperature
SO_2_	0.46*^a^*			

NO_2_	0.64*^a^*	0.65*^a^*		

Temperature	0.04	-0.68*^a^*	-0.28*^a^*	

Humidity	0.28*^a^*	-0.12*^a^*	0.25*^a^*	0.21*^a^*

*^a ^*P < 0.05

**Figure 2:**
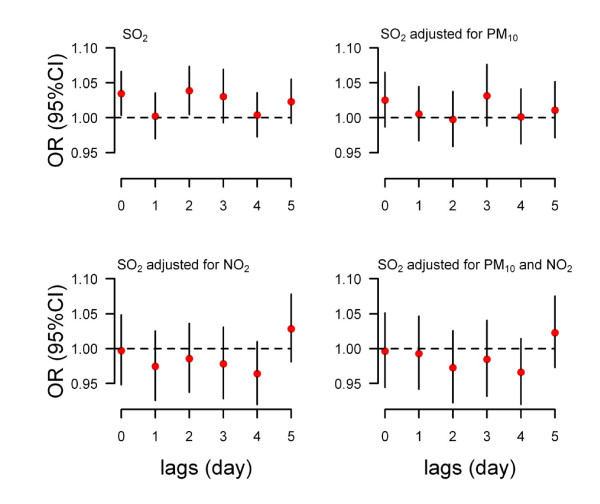
**The association between a 10 ug/m^3 ^increase in SO_2 _and daily emergency hospital visits for hypertension at lag days 0 to 5 in single pollutant and multiple pollutants models (time-stratified case-crossover controlling temperature and relative humidity)**.

**Figure 3:**
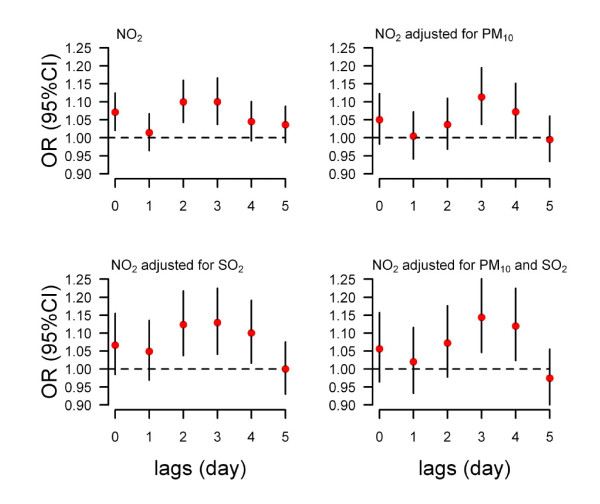
**The association between a 10 ug/m^3 ^increase in NO_2 _and daily emergency hospital visits for hypertension at lag days 0 to 5 in single pollutant and multiple pollutants models (time-stratified case-crossover controlling temperature and relative humidity)**.

A 10 μg/m^3 ^increase in SO_2 _was significantly associated with EHVs at lags of 0 and 2 days (Top-left of figure 2), while NO_2 _was significantly associated with EHVs at lags of 0, 2 and 3 days (Top-left of figure 3). According to the OR values and the 95% confidence intervals, we chose the current day and three days after exposure as the hazard period for SO_2 _and NO_2_, respectively. The ORs were 1.037 (95% CI: 1.004-1.071) for SO_2 _and 1.101 (95% CI: 1.038-1.168) for NO_2 _(Table 3).

**Table 3:** Odds ratios for daily emergency hospital visits for hypertension for a 10 μg/m^3 ^increase in air pollutants (Results from a time-stratified case-crossover for single pollutant and multiple pollutants models) *^a^*

Air pollutants	OR	95% CI	
		
		lower	upper
SO_2_	1.037*^b^*	1.004	1.071

+PM_10_	1.025	0.987	1.065

+NO_2_	0.997	0.949	1.048

+PM_10_+NO_2_	0.997	0.945	1.051

NO_2_	1.101*^b^*	1.038	1.168

+PM_10_	1.114*^b^*	1.037	1.195

+SO_2_	1.130*^b^*	1.041	1.225

+PM_10_+SO_2_	1.144*^b^*	1.046	1.251

*^a ^*ORs of SO_2 _are at lag 0 day; ORs of NO_2 _are at lag 3 day; OR: odds ratio; CI: confidence interval;

*^b ^*P < 0.05

The results of multiple pollutant models are shown in Figure 2, Figure 3 and Table 3. For SO_2_, after controlling PM_10_, NO_2_, or both PM_10 _and NO_2_, the ORs were no longer statistically significant and the means were lower than the single pollutant model. After adjusting for PM_10_, SO_2_, or both SO_2 _and PM_10_, the mean effects of NO_2 _at lag 3 days were higher than the single pollutant model.

## Discussion

This is the first study to examine the association between gaseous air pollution and EHVs for hypertension in Beijing, China. The time-stratified case-crossover was used to examine the relationship between gaseous air pollution and EHVs for hypertension. We found that gaseous air pollution had a significant impact on the EHVs visits for hypertension. An increase of 10 μg/m^3 ^in levels of SO_2 _and NO_2 _were associated with an increase of 3.7% (95% CI: 0.4% - 7.1%) and 10.1% (95% CI: 3.8% - 16.8%) in EHVs for hypertension, respectively. Figure 1 illustrates that the concentrations of SO_2 _and NO_2 _were remarkably higher in winter, which may be due to the increased use of heating in winter. However, we controlled for this seasonal change using the case-crossover. The major sources of SO_2 _and NO_2 _in Beijing were heating and industrial sources. Besides, urban NO_2 _levels are closely related to traffic emissions [28].

In order to understand if there were any short-term delays between gaseous air pollution and hypertension, the lags of 0 to 5 days were examined in the single air pollutant models. The results show that the adverse effects of SO_2 _on EHVs for hypertension were statistically significant at lags of 0 and lag 2 days, while the impact of NO_2 _was significant at lags of 0, 2, and 3 days. In a previous study, we examined the relationship between air pollution and EHVs for cardiovascular diseases from the same hospital between 2004 and 2006 and found that after adjusting for temperature and relative humidity, the ORs for EHVs for cardiovascular diseases were 1.014 (95% CI: 1.004-1.024) and 1.016 (95% CI: 1.003-1.029) for a 10 μg/m^3 ^increase in levels of SO_2 _or NO_2 _at lag 0 day, respectively [1]. This study suggests that the gaseous air pollution has a lagged effect on EHVs for hypertension.

In the multiple pollutant models, the OR values of SO_2 _were lower than the single pollutant models, particularly after controlling for NO_2_. Conversely, the OR values of NO_2 _were higher than the single pollutant models. Previous research [1,29] on the association between air pollution and mortality and EHVs for cardiovascular diseases in Beijing showed the same results. There may be some co-linearity between SO_2 _and NO_2 _(Figure 1 and Table 2).

Studies have examined the potential biological mechanisms that help explain the effects of SO_2 _and NO_2 _on the cardiovascular system. As ambient SO_2 _concentrations rise, the SO_2 _concentration in blood and other tissues of the body increase [30]. When SO_2 _is inhaled the lipid peroxidation level in mice is raised. SO_2 _at all tested concentrations significantly decreased activities of superoxide dismutase in mice of both sexes, as well as that of glutathione peroxidase from male mice [31].

Some studies suggest that elevated levels of NO_2 _could increase the number of EHVs for cardiovascular disease [5,6]. Studies on the biological mechanism found that increased NO_2 _is associated with increased plasma fibrinogen, ventricular arrhythmia and ventricular tachycardia [32-34]. However, recently, Langrish et al. [35] found that inhalation of NO_2 _did not impair vascular vasomotor or fibrinolytic function in man.

Previous studies found that gaseous air pollutants have adverse effects on heart rate variability (HRV) which reflects cardiac autonomic function. Min et al. [36] measured HRV among community residents in Korea to study the effects of PM_10_, SO_2_, and NO_2 _on cardiac autonomic function, and found that exposure to PM_10_, SO_2_, and NO_2 _resulted in reduced HRV, significant decreases in the standard deviation of the normal to normal interval (SDNN) and low frequency (LF) domain effect, and the effect was sustained for twelve hours. Routledge et al. [37] found that SO_2 _exposure resulted in a significant reduction in HRV markers of cardiac vagal control at four hours in healthy people, but no changes were found in patients with stable angina. de Paula Santos et al. [19] observed that an inter-quartile range increase of 9.6 μg/m^3 ^in level of SO_2 _was negatively associated with SDNN of -7.93 ms (95% CI: -15.3, - 0.6). Chan et al. [38] carried out a panel study to examine the association between NO_2 _and HRV in a susceptible population with coronary heart disease (CHD) or more than one major CHD risk factor, and found that an increase of 10 ppb in NO_2 _was associated with 1.5-2.4% decreases in SDNN, and for each 10 ppb increase in NO_2 _the LF was decreased by 2.2-2.5%.

Much research has been carried out to look at the influence of gaseous air pollution on mortality and morbidity for cardiovascular disease and shown that the increased concentrations of gaseous air pollution can impact on population health. Kan and Chen [39] applied a case-crossover to evaluate the relation between air pollution and daily mortality in Shanghai. The results showed that an increase of 10 μg/m^3 ^in the levels of SO_2 _and NO_2 _had a relative risk of 1.017 (95% CI: 1.009-1.026) and 1.024 (95% CI: 1.011-1.036) for cardiovascular mortality. D'Ippoliti et al. [10] conducted a time-stratified case-crossover to explore the relationship between urban air pollutants and hospital admissions for acute myocardial infarction in Rome, and showed that each 10 μg/m^3 ^increase of NO_2 _was associated with a relative risk of 1.026 (95%CI: 1.002-1.052) for hospital admissions for acute myocardial infarction. Grazuleviciene et al. [40] carried out a population-based case-control study among men aged 25-64 years residing in Kaunas, to explore the relationship between long-term exposure to NO_2 _and myocardial infarction. Results suggested that urban NO_2 _pollution increased the risk of myocardial infarction.

This study has three strengths. Firstly, to our knowledge, it is the first epidemiological study which has specifically explored the relationship between gaseous air pollution and EHVs for hypertension. Secondly, a relative large sample size was used, with a considerable daily variance of exposure and outcomes. Finally, EHVs are a good indicator of the acute effects of air pollution, because when people in China with cardiovascular disease feel uncomfortable, the first choice they usually make is to visit the hospital emergency department.

This study also has some limitations. The cases were only selected from one hospital. Although the patients lived near the hospital, it still cannot control their hospital selection. Outdoor average concentrations of PM_10_, SO_2 _and NO_2 _were collected from fixed sites, but the data on individual exposure were unavailable. There might be misclassification bias for coding EHVs, because the diagnosis of EHVs for hypertension can never be 100% correct. Socioeconomic factors were not considered, as we could not get the patients' data on socioeconomics. The study was only conducted in the urban city of Beijing, and therefore the generalisability of the results is limited.

## Conclusion

We found that elevated concentrations of gaseous air pollutants were associated with EHVs for hypertension in Beijing, China. The findings provide additional information about the health effects of air pollution, and may have implications for planning local environmental protection and public health interventions.

## Abbreviations

EHVs: emergency hospital visits; SBP: systolic blood pressure; DBP: diastolic blood pressure; ICD10: International Classification of Disease, tenth revision; SO_2_: sulfur dioxide; NO_2_: nitrogen dioxide; PM_10_: particulate matter less than 10 μm in aerodynamic diameter; OR: odds ratio; CI: confidence interval; HRV: heart rate variability; SDNN: standard deviation of the normal to normal interval; LF: low frequency; CHD: coronary heart disease; SD: standard deviation;

## Competing interests

The authors declare that they have no competing interests.

## Authors' contributions

YMG conceived and coordinated the study, performed data analysis and drafted the manuscript; SLT and SSL contributed to study design, reviewed and edited the manuscript; AGB contributed to statistical analysis, reviewed and edited the manuscript; WWY and YSZ contributed to review and edit the manuscript. XCP provided air pollution information and health data, and edited the manuscript; All authors have read and approved the final manuscript.

## References

[B1] Guo Y Jia Y Pan X Liu L Wichmann HE The association between fine particulate air pollution and hospital emergency room visits for cardiovascular diseases in Beijing, China Sci Total Environ 2009 407 4826 4830 10.1016/j.scitotenv.2009.05.022 19501385

[B2] Simkhovich BZ Kleinman MT Kloner RA Particulate air pollution and coronary heart disease Curr Opin Cardiol 2009 24 604 609 10.1097/HCO.0b013e32833161e5 19696664

[B3] Oudin A Stroh E Stromberg U Jakobsson K Bjork J Long-term exposure to air pollution and hospital admissions for ischemic stroke. A register-based case-control study using modelled NO(x) as exposure proxy BMC Public Health 2009 9 301 10.1186/1471-2458-9-301 19691845 PMC2736944

[B4] Halonen JI Lanki T Yli-Tuomi T Tiittanen P Kulmala M Pekkanen J Particulate air pollution and acute cardiorespiratory hospital admissions and mortality among the elderly Epidemiology 2009 20 143 153 10.1097/EDE.0b013e31818c7237 19234403

[B5] Santos UP Terra-Filho M Lin CA Pereira LA Vieira TC Saldiva PH Braga AL Cardiac arrhythmia emergency room visits and environmental air pollution in Sao Paulo, Brazil J Epidemiol Community Health 2008 62 267 272 10.1136/jech.2006.058123 18272743

[B6] Szyszkowicz M Ambient air pollution and daily emergency department visits for ischemic stroke in Edmonton, Canada Int J Occup Med Environ Health 2008 21 295 300 10.2478/v10001-008-0029-5 19158072

[B7] Slaughter JC Kim E Sheppard L Sullivan JH Larson TV Claiborn C Association between particulate matter and emergency room visits, hospital admissions and mortality in Spokane, Washington J Expo Anal Environ Epidemiol 2005 15 153 159 10.1038/sj.jea.7500382 15187986

[B8] Kan H Heiss G Rose KM Whitsel EA Lurmann F London SJ Prospective analysis of traffic exposure as a risk factor for incident coronary heart disease: the Atherosclerosis Risk in Communities (ARIC) study Environ Health Perspect 2008 116 1463 1468 10.1289/ehp.11290 19057697 PMC2592264

[B9] Chiu HF Yang CY Air pollution and emergency room visits for arrhythmias: are there potentially sensitive groups? J Toxicol Environ Health A 2009 72 817 823 10.1080/15287390902800405 19557609

[B10] D'Ippoliti D Forastiere F Ancona C Agabiti N Fusco D Michelozzi P Perucci CA Air pollution and myocardial infarction in Rome: a case-crossover analysis Epidemiology 2003 14 528 535 10.1097/01.ede.0000082046.22919.72 14501267

[B11] Lee IM Tsai SS Ho CK Chiu HF Wu TN Yang CY Air pollution and hospital admissions for congestive heart failure: are there potentially sensitive groups? Environ Res 2008 108 348 353 10.1016/j.envres.2008.07.024 18786668

[B12] Wellenius GA Schwartz J Mittleman MA Particulate air pollution and hospital admissions for congestive heart failure in seven United States cities Am J Cardiol 2006 97 404 408 10.1016/j.amjcard.2005.08.061 16442405

[B13] Guo Y Tong S Zhang Y Barnett AG Jia Y Pan X The relationship between particulate air pollution and emergency hospital visits for hypertension in Beijing, China Sci Total Environ 2010 408 4446 4450 10.1016/j.scitotenv.2010.06.042 20638709

[B14] Lopez AD Mathers CD Ezzati M Jamison DT Murray CJ Global and regional burden of disease and risk factors, 2001: systematic analysis of population health data Lancet 2006 367 1747 1757 10.1016/S0140-6736(06)68770-9 16731270

[B15] Levy D Larson MG Vasan RS Kannel WB Ho KK The progression from hypertension to congestive heart failure JAMA 1996 275 1557 1562 10.1001/jama.275.20.1557 8622246

[B16] Wexler RK Treatment of hypertension critical in reducing morbidity and mortality J Am Board Fam Med 2007 20 322 10.3122/jabfm.2007.03.070002 17478670

[B17] Xu L Wang S Wang YX Wang YS Jonas JB Prevalence of arterial hypertension in the adult population in rural and urban China: the Beijing eye study Am J Hypertens 2008 21 1117 1123 10.1038/ajh.2008.247 18670415

[B18] Ibald-Mulli A Stieber J Wichmann HE Koenig W Peters A Effects of air pollution on blood pressure: a population-based approach Am J Public Health 2001 91 571 577 10.2105/AJPH.91.4.571 11291368 PMC1446632

[B19] de Paula Santos U Braga AL Giorgi DM Pereira LA Grupi CJ Lin CA Bussacos MA Zanetta DM do Nascimento Saldiva PH Filho MT Effects of air pollution on blood pressure and heart rate variability: a panel study of vehicular traffic controllers in the city of Sao Paulo, Brazil Eur Heart J 2005 26 193 200 10.1093/eurheartj/ehi035 15618077

[B20] Choi JH Xu QS Park SY Kim JH Hwang SS Lee KH Lee HJ Hong YC Seasonal variation of effect of air pollution on blood pressure J Epidemiol Community Health 2007 61 314 318 10.1136/jech.2006.049205 17372291 PMC2652940

[B21] Auchincloss AH Diez Roux AV Dvonch JT Brown PL Barr RG Daviglus ML Goff DC Kaufman JD O'Neill MS Associations between recent exposure to ambient fine particulate matter and blood pressure in the Multi-ethnic Study of Atherosclerosis (MESA) Environ Health Perspect 2008 116 486 491 10.1289/ehp.116-a486 18414631 PMC2291007

[B22] Lee JT Schwartz J Reanalysis of the effects of air pollution on daily mortality in Seoul, Korea: A case-crossover design Environ Health Perspect 1999 107 633 636 10.1289/ehp.99107633 10417360 PMC1566500

[B23] Basu R Dominici F Samet JM Temperature and mortality among the elderly in the United States: a comparison of epidemiologic methods Epidemiology 2005 16 58 66 10.1097/01.ede.0000147117.88386.fe 15613946

[B24] Armstrong B Models for the relationship between ambient temperature and daily mortality Epidemiology 2006 17 624 631 10.1097/01.ede.0000239732.50999.8f 17028505

[B25] Braga AL Zanobetti A Schwartz J The time course of weather-related deaths Epidemiology 2001 12 662 667 10.1097/00001648-200111000-00014 11679794

[B26] Barnett AG Dobson AJ Analysing Seasonal Health Data 2010 Berlin, Heidelberg: Springer

[B27] Barnett AG Baker PJ Dobson AJ season: Analysing Seasonal Data R Functions R package version 0.2-5 2010

[B28] Meng ZY Ding GA Xu XB Xu XD Yu HQ Wang SF Vertical distributions of SO(2) and NO(2) in the lower atmosphere in Beijing urban areas, China Sci Total Environ 2008 390 456 465 10.1016/j.scitotenv.2007.10.012 18037476

[B29] Yang M Pan X Time-series analysis of air pollution and cardiovascular mortality in Beijing, China J Environ Health 2008 25 294 297

[B30] Etlik O Tomur A Kutman MN Yorukan S Duman O The effects of sulfur dioxide inhalation and antioxidant vitamins on red blood cell lipoperoxidation Environ Res 1995 71 25 28 10.1006/enrs.1995.1063 8757235

[B31] Meng Z Qin G Zhang B Geng H Bai Q Bai W Liu C Oxidative damage of sulfur dioxide inhalation on lungs and hearts of mice Environ Res 2003 93 285 292 10.1016/S0013-9351(03)00045-8 14615239

[B32] Pekkanen J Brunner EJ Anderson HR Tiittanen P Atkinson RW Daily concentrations of air pollution and plasma fibrinogen in London Occup Environ Med 2000 57 818 822 10.1136/oem.57.12.818 11077010 PMC1739901

[B33] Peters A Dockery DW Muller JE Mittleman MA Increased particulate air pollution and the triggering of myocardial infarction Circulation 2001 103 2810 2815 11401937 10.1161/01.cir.103.23.2810

[B34] Schwartz J Air pollution and blood markers of cardiovascular risk Environ Health Perspect 2001 109 405 409 10.2307/3434788 11427390 PMC1240558

[B35] Langrish JP Lundback M Barath S Soderberg S Mills NL Newby DE Sandstrom T Blomberg A Exposure to nitrogen dioxide is not associated with vascular dysfunction in man Inhal Toxicol 2010 22 192 198 10.3109/08958370903144105 20047363

[B36] Min KB Min JY Cho SI Paek D The relationship between air pollutants and heart-rate variability among community residents in Korea Inhal Toxicol 2008 20 435 444 10.1080/08958370801903834 18302051

[B37] Routledge HC Manney S Harrison RM Ayres JG Townend JN Effect of inhaled sulphur dioxide and carbon particles on heart rate variability and markers of inflammation and coagulation in human subjects Heart 2006 92 220 227 10.1136/hrt.2004.051672 15923279 PMC1860755

[B38] Chan CC Chuang KJ Su TC Lin LY Association between nitrogen dioxide and heart rate variability in a susceptible population Eur J Cardiovasc Prev Rehabil 2005 12 580 586 10.1097/00149831-200512000-00011 16319549

[B39] Kan H Chen B A case-crossover analysis of air pollution and daily mortality in Shanghai J Occup Health 2003 45 119 124 10.1539/joh.45.119 14646303

[B40] Grazuleviciene R Maroziene L Dulskiene V Malinauskiene V Azaraviciene A Laurinaviciene D Jankauskiene K Exposure to urban nitrogen dioxide pollution and the risk of myocardial infarction Scand J Work Environ Health 2004 30 293 298 15458012 10.5271/sjweh.797

